# Annotated genome assemblies of two temperate North American dung beetles, *Canthon chalcites* and *Phanaeus vindex*

**DOI:** 10.1093/g3journal/jkag194

**Published:** 2026-08-05

**Authors:** Gwendolyn F Stark, Karin R L van der Burg, Alexander J Coverley, Katie E Marshall, Kimberly S Sheldon

**Affiliations:** Department of Ecology and Evolutionary Biology, University of Tennessee, Knoxville, TN 37996, United States; Department of Biological Sciences, Clemson University, Clemson, SC 29631, United States; Department of Zoology, University of British Columbia, Vancouver, Bc, Canada V6T 1Z4; Department of Zoology, University of British Columbia, Vancouver, Bc, Canada V6T 1Z4; Department of Ecology and Evolutionary Biology, University of Tennessee, Knoxville, TN 37996, United States

**Keywords:** dung beetle, temperate, genome assembly, genome annotation

## Abstract

Dung beetles serve as cultivators of their natural habitats, improving soil health and functions in both natural and anthropogenic environments. Despite their ecological importance, whole genome sequences for Scarabaeinae are limited. Here, we present the draft annotated genome assemblies for 2 temperate species of North American dung beetles collected from eastern Tennessee: *Canthon chalcites* and *Phanaeus vindex*. Both genome assemblies were generated from PacBio long reads and have high completeness, with BUSCO scores of 98.1% and 98.6% for *C. chalcites* and *P. vindex*, respectively. For *C. chalcites*, the BRAKER3 pipeline predicted 12,799 genes, and the gene set was 93.7% complete. For *P. vindex*, the BRAKER3 predicted 12,252 genes, and the gene set was 94.9% complete. From the annotated gene sets, orthologous protein sequence analyses among *C. chalcites*, *P. vindex*, the dung beetle species *Onthophagus taurus*, and the more evolutionarily distant beetle *Tribolium castaneum* indicated that there are 260 unique protein clusters for *C. chalcites* and 210 unique protein clusters for *P. vindex.* These 2 draft genomes provide valuable data for comparative genomics, evolution, and phylogenic studies for dung beetle species.

## Introduction

Dung beetles (Scarabaeidae, Scarabaeinae) are the recyclers of the insect world, playing a pivotal role in multiple ecosystem functions. Dung beetles bury feces underground for feeding and breeding purposes, and in turn they remove animal waste from the soil surface (deCastro-Arrazola et al. 2022). These activities aid in mineralization of organic matter by enhancing soil microbial communities (Wassmer and Armstrong 2023), reducing the development of greenhouse gases (Wassmer and Armstrong 2023), and promoting soil aeration (Nichols et al. 2008). It has been estimated that there are more than 6,200 dung beetle species (Tarasov and Génier 2015; Tarasov and Dimitrov 2016); however, there are currently only 26 Scarabaeinae genome assemblies on NCBI, which accounts for genome sequences of less than 1% of estimated dung beetle species. Thus, research on development and physiology would benefit from additional molecular resources for the Scarabaeinae group.

We sequenced the genomes of 2 temperate dung beetles, *Phanaeus vindex* Macleay, 1819, and *Canthon chalcites* Haldeman, 1843, collected from eastern Tennessee, United States. Currently, bioinventories of dung beetles from this region of Appalachia are rare and incomplete (but see Mamantov and Sheldon 2023). *Canthon chalcites* is a large dung beetle (13 to 21 mm) in the telecoprid guild (rollers) (Mamantov and Sheldon 2023). It is native to the eastern United States and is active from June to September, with maximum activity occurring in mid-July. *Phanaeus vindex* is also native to North America, belonging to the paracoprid guild (tunnelers) (Mamantov and Sheldon 2023). Similar to *C*. *chalcites*, *P. vindex* is active from June to September, with activity peaking in late August (Kirkpatrick and Sheldon 2022; Mamantov and Sheldon 2023).

As ectotherms, dung beetles are sensitive to ambient temperatures (Bale and Hayward 2010). In temperate zones, like eastern Tennessee, temperatures can have large daily fluctuations, sometimes up to 20 °C (Marshall and Sinclair 2012). Unfavorable growth temperatures can impact the development and fitness of beetles in early life stages (Holley and Andrew 2019; Fleming et al. 2021), but dung beetles are capable of significant behavioral and physiological plasticity to overcome temperature fluctuations (Carter and Sheldon 2020; Sheldon et al. 2020; Macagno et al. 2018). This makes them an important group for understanding the molecular mechanisms of both phenotypic and behavioral plasticity, and genomic resources from *C. chalcites* and *P. vindex* will aid in better understanding the evolutionary strategies of this clade (Matthews and Vosshall 2020; Killeffer et al. 2025).

While both *C. chalcites* and *P. vindex* reside in the temperate climate of eastern Tennessee, *P. vindex* males display major–minor dimorphism, while *C. chalcites* males do not (Killeffer et al. 2025). Male dung beetles with major–minor dimorphism have differing reproductive strategies, where major males develop large horns that they use to compete for and guard breeding females (Moczek and Emlen 2000). In contrast, minor males are horn-less and use less aggressive sneaking tactics to gain access to females (Oliveira et al. 2008). Minor males also have a greater investment in reproductive organs, which may be advantageous when it comes to sperm competition with major males (Killeffer et al. 2025). Therefore, the genomes of *C. chalcites* and *P. vindex* will serve as important resources for studies focusing on development and the evolution of phenotypic plasticity and dimorphism in dung beetles.

## Methods

### Beetle collection

In 2021, we collected beetles with baited pitfall traps at Seven Islands State Birding Park in Kodak, Tennessee (35°57′143″N, −83°41′221″W; permit number TDEC-2020-030). We transported the beetles to the lab in 950-mL plastic containers with moist paper towels. Beetles were kept in small, species-separated groups at room temperature (22 °C) and fed cow dung *ad libitum* for 7 to 14 d. For shipping, we packed plastic containers (∼113 g) with paper towels to house 6 to 8 beetles of the same species. We then placed the plastic containers in a Styrofoam cooler with small ice packs at the bottom and shipped them overnight to Washington State, after which the beetles were imported into Canada (permit number CFIA 5256 from Canadian Food Inspection Agency).

### DNA extraction

We extracted DNA using the Monarch Genomic DNA Purification Kit from New England Biolabs (T3010S), with slight modifications to the protocol. In short, for each species, we dissected 1 adult male and removed the soft tissue from the head and thorax. We avoided the abdomen due to potential contamination of gut contents. A total of 25 mg of tissue was placed in 200-uL lysis buffer in a 2-mL Dounce homogenizer for gentle tissue disruption. After douncing, we transferred the tissue to a 1.5-mL Eppendorf tube with a wide-mouth pipette tip. We added 10-uL Proteinase K and mixed samples by flicking the tube. We allowed lysis to continue overnight at 56 °C on a tube rotator.

### RNA extraction and sequencing

We weighed and acclimated female beetles of both species to 22 ± 2 °C in a 12:12 L:D cycle for 11 d and fed them sterilized cow dung *ad libitum* from animals that had not been treated with antibiotics or antiparasitic medication. We euthanized beetles of both species using low temperature and stored them at −80 °C before shipping to the University of British Columbia (UBC) for processing. At UBC, we removed *C. chalcites* from the freezer and dissected them before thawing occurred to examine the reproductive organs, and those with an aedeagus were discarded; we used only females for further processing.

For each transcriptome library, we cut females of both species with scissors, submerged a single beetle in 1-mL TRIzol reagent, and then homogenized it using 1-mm zircon beads in a Storm 24 bullet blender (Next Advance, Averill Park, USA) for 10 min. We centrifuged the samples at 12,000 × *g* for 10 min at 2 °C, inverted 5 times, and spun down for a further 30 s. We transferred an aliquot of 200 µL of the supernatant to the tubes and diluted with TRIzol up to a total volume of 1 mL. To conduct the phase separation, we added 200 µL of chloroform to each sample and then vigorously shook the samples. We then centrifuged the samples for 15 min at 12,000 × *g* and 2 °C. We then extracted 300 µL of the upper phase and added 250 µL of ice-cold molecular grade isopropanol. Next, we centrifuged the upper phases for 10 min at 12,000 × *g* and 2 °C, discarded the supernatant, and then added 250 µL of 100% ethanol to wash the pellet. We then centrifuged the samples at 7500 × *g* for 5 min and repeated the ethanol pellet washing 2 more times. Finally, we removed the ethanol and air-dried the samples for 7 min before diluting in 50 µL of molecular grade water. We vortexed the samples and then incubated them in a water bath at 55 °C for 10 min to dissolve the pellet. We assessed the sample quality using a Thermo Fisher NanoDrop 2000 Spectrophotometer, and we selected the 6 highest quality samples. We sent 20 µL per sample, at a concentration of 100 ng/µL, on dry ice to Génome Québec for library preparation and sequencing. Génome Québec used the extracted RNA to prepare libraries using an NEBNext polyA enrichment kit, followed by paired-end sequencing with 100-bp reads on a Illumina NovaSeq system at a depth of 40 M reads per sample.

### PacBio long-read sequencing and read processing

We sent extracted DNA to Maryland Genomics for library preparation and sequencing. For each specimen, 1 PacBio HiFi library was prepared and sequenced on 1 PacBio Sequel II 8M SMRT Cell run (CCS/HiFi mode—30-h movie). We assessed the quality of the PacBio circular consensus reads (ccs) using NanoPlot (v. 1.44.1) (De Coster and Rademakers 2023). For both genomes, 100% of the raw reads had Q scores > 20, so all reads were used in the assembly process (Supplementary Table 1). Quality read scores and raw read NanoPlot statistics for *C. chalcites* and *P. vindex* can be found in Supplementary Table 1.

### Initial genome assembly

For genome assembly, we used high-quality ccs PacBio long reads (all reads Q > 20). We assembled genomes using hifiasm (v 0.19.5), with default parameters enabled. For downstream analysis, we used the primary haplotype-purged.ctg file. For genome assembly statistics and quality checks, we utilized QUAST (v. 5.0.2) (Gurevich et al. 2013), BUSCO (v.5.2.2) and BUSCO (v.6) (Simao et al. 2015; Seppey et al. 2019; Manni et al. 2021). Initially, we ran BUSCO with the insecta_obd10 lineage, and all other parameters were left as default. We used the BUSCO insecta_obd10 outputs in downstream contamination checks using BlobToolKit. The insecta_obd12 lineage was released in July 2025, and we used it to generate statistics for the initial and final genome assemblies as well as the gene annotation sets.

### Contamination screen

For the initial contamination screen, we used BlobToolKit (v.4.4.1) (Challis et al. 2020). We split the assembly file for each genome into separate fasta files using seqkit (Shen et al. 2016), with 100 contigs per file. Each FASTA file was searched against the NCBI nr nucleotide database using BLAST (v. 2.14.1) with the following parameters: -outfmt “6 qseqid staxids bitscore std”, e-value of 1e-6, and max_target_seqs 10. We then concatenated all BLAST output files together and used them for downstream BlobToolKit analysis. For contig coverage, we mapped the raw PacBio ccs reads to the genomes using minimap2 (v. 2.28) (Li 2018), and we indexed the BAM output file with samtools (v1.20). We also incorporated BUSCO (v.5.2.2) scores in the BlobToolKit contamination check. We used the BlobToolKit v.4.4.1 online viewer to filter out contaminant contigs from the assemblies.

Initially, both genomes had a large number of no-hit contigs from the BLAST results. Because of this, we reran the nucleotide BLAST for the no-hit contigs with a less stringent e-value of 1e-03. To determine which contigs would be included in the final assemblies, we considered both taxonomy of the contigs and contigs with insecta_obd10 BUSCO hits. For the *P. vindex* genome, we included all contigs taxonomically classified as arthropod in the final assembly. For the *C. chalcites* genome, we included all arthropod contigs, 3 “no-hit” contigs, and 1 Chordata contig in the final assembly. The included no-hit and Chordata contigs had BUSCO hits, which we manually ran through NCBI BLASTx (ClusteredNR database, expect value of 0.05) to determine if these contigs had other insect hits (Supplementary Table 2).

For a final contamination check, we ran both genomes through the NCBI Foreign Contamination Screen software and manually trimmed or removed any remaining contaminant sequences from the final assemblies. We removed from the assembly any contigs <1,000 bp in size after the manual trimming. We then ran the genomes through BUSCO (v.6) and QUAST (v.5.0.2) to get final completeness statistics.

### Purging haplotype contigs

After removing initial contaminant contigs from the assemblies with BlobToolKit, we ran the genomes through purge_dups (v.1.2.5) to remove any potential remaining duplicate haplotype contigs (Guan et al. 2020). We mapped reads with minimap (Li 2018) (v. 2.24) with –asm alignment on, and we used the .pfz file as the input file for purge_dups. We ran the contigs’ output in the purged.fa file through BUSCO (v.5.2.2) to determine the duplication of universal single-copy orthologs and to get genome completeness scores.

### Repeat annotation and masking

Prior to gene annotation, we used RepeatModeler (v 2.0.3) (Flynn et al. 2020) to build a custom repeat library for each genome. In RepeatModeler, we used rmblast (v 2.10.0) as the search engine and enabled –LTRStruct, keeping all other parameters as default. We used the repeat library generated by RepeatModeler along with the respective genome as input for RepeatMasker (v 4.1.2-p1), with –xsmall soft masking of the genomes enabled and rmblast (v 2.10.0) as the search engine. The masked genome file produced from RepeatMasker was our input genome for BRAKER3 annotation.

### Mitochondrial genome removal

We used MitoHiFi (Uliano-Silva et al. 2023) to identify putative mitochondrial contigs in the genome assemblies, which we then manually verified using BLAST to confirm their mitochondrial origins. To see if candidate contigs were complete mitochondrial genomes, we annotated with MITOS2, using the invertebrate code and metozoa63 as a reference. To remove mitochondrial contigs from the genomes, we used seqkit (v 2.5.1). For *C. chalcites*, we completely removed 1 contig from the assembly as it had a complete set of mitochondrial genes. Two contigs in the *P. vindex* genome were identified as mitochondrial by MitoHifi. One of these contigs was noted as circular, and we removed it from the assembly. The other contig (ptg000379l) was 58,511 bp long, but only had mitochondrial genes from regions 44,856 to 58,511 bp Because of this, we only removed the mitochondrial region of this contig from the genome assembly. Both mitochondrial regions in *P. vindex* were missing the gene *trnK*. Mitochondrial sequences for both genomes can be found in Supplementary Files 1 and 2.

### Transcriptome preparation for genome annotation

We trimmed all RNA-seq fastq reads for quality using fastp (v 0.24.0) (Chen et al. 2018; Chen 2023). For all fastp runs, we set the following parameters: –detect_adapter_for_pe, –qualified_quality_phred 20, cut_tail_mean_quality 20, trim_front –10, –trim_poly_g, and –length_required 30. After trimming and quality filtering, we checked RNA reads for quality using FastQC (v 0.12.1) and MultiQC (v.1.29). We aligned all quality trimmed transcriptomic libraries against their respective genomes using the splice-aware aligner HISAT2 (v 2.2.1) (Kim et al. 2019). For HISAT2 alignment, we enabled –dta for splice-aware aligning and kept all other parameters as default. We sorted the resulting BAM files with samtools (v1.20) to prepare for transcript assembly. Using StringTie (v 2.2.3) (Pertea et al. 2015), we assembled transcripts from the sorted bam files, producing .gtf annotation files that we converted to fasta format using gffread (v.0.12.3) (Pertea and Pertea 2020).

### Gene prediction and annotation

We used BRAKER3 to annotate the genomes because it utilizes RNA-seq and protein data to train Augustus and GeneMark, resulting in reliable gene predictions (Gabriel et al. 2024). For RNA-seq evidence, we concatenated the HISAT2 transcriptomic alignment bam files for each respective genome and then used them as input for BRAKER3. We used the soft-masked version of both genomes (completed with RepeatMasker) as genomic input. For the protein database input, we used the reviewed insect protein sequence database on Swiss-Prot (tax id = 50557) as well as the proteomes from the beetles *Tribolium castaneum* and *Oryctes borbonicus*. We concatenated the protein sequences from these sources and used seqkit (v 2.5.1) to discard any duplicated protein sequences. We ran BRAKER3 in ETP mode and kept all other parameters as default. We converted the resulting braker.gtf file to gff3 format using the AGAT gff toolkit (Dainat) and used InterProScan (v. 5.73-104.0) (Quevillon et al. 2005) to find functional domains for the predicted genes.

Due to the NCBI foreign contamination screen, some contig IDs and coordinates in the final gff files had to be manually corrected for both genomes. We manually removed 2 genes from the *C. chalcites* gff annotation file because they spanned a region that was split due to adapter contamination. We kept all genes for *P. vindex*.

### Comparative genomic analyses

We used OrthoVenn3 (Sun et al. 2023) to investigate the orthologous relationships between our 2 newly annotated species, as well as the dung beetle *Onthophagus taurus*, and a more divergent Coleoptera species, *T. castaneum*. We ran the orthologous analysis on the predicted protein sequences, including isoforms, from all 4 species, using the OrthoMCL algorithm, with an E-value of 1E-3, and with the annotation, protein similarity, and cluster relationship network enabled. We also completed phylogenetic analyses, gene family contractions, and expansion analyses. For the phylogenetic analyses, we used the JTT + CAT model. For the expansion and contractions analysis, we used the divergence time between *T. castaneum* and *O. taurus* (256.47 MYA, estimated from the Time Tree of Life) as input.

Since we were interested in the proteins that expanded or were more represented in *C. chalcites* and *P. vindex*, we used the GO enrichment output produced by OrthoVenn3 for the expansion analysis as input for rrvgo (Sayols 2023). We used rrvgo to help us further interpret GO term similarities and reduce redundancy in our GO enrichment lists by grouping GO terms that were part of the biological processes ontology by semantic similarity. To group our GO terms involved in biological processes, we used the *Drosophila melanogaster* database (org.Dm.eg.db). For sematic similarity analysis, we left out 11 GO terms for *C. chalcites* and 7 GO terms for *P. vindex* involved in biological processes that did not match the *D. melanogaster* database.

## Results

### *Canthon chalcites* genome assembly

Initially, the raw *C. chalcites* genome had a cumulative length of ∼639.31 Mbp and consisted of 2,435 contigs. After removing all contaminant contigs, mitochondrial contigs, and any additional haplotype contigs, the final *C. chalcites* nuclear genome had a cumulative length of ∼556.82 Mbp and consisted of 834 contigs with an L50 of 61. The N50 was ∼2.28 Mbp, and the GC content was 33.37%. All QUAST statistics for the final *C. chalcites* assembly can be found in Table 1. For the final assembly, BUSCO scores showed the genome to be 98.1% complete, with a single-copy completeness score of 95.2% with 0% gaps. For the final assembly, there was a 2.9% BUSCO duplication score, which was an improvement over the initial assembly that had a duplication score of 8.5% (Table 2). The final genome sequencing coverage was ∼20×.

**Table 1.** Final genome assembly statistics generated by QUAST for *C. chalcites* and *P. vindex*.

	*C. chalcites*	*P. vindex*
# contigs (≥0 bp)	834	384
# contigs (≥1,000 bp)	834	384
# contigs (≥5,000 bp)	834	383
# contigs (≥10,000 bp)	800	378
# contigs (≥25,000 bp)	653	301
# contigs (≥50,000 bp)	561	197
Total length (≥0 bp)	555,823,926	409,254,878
Total length (≥1,000 bp)	555,823,926	409,254,878
Total length (≥5,000 bp)	555,823,926	409,252,696
Total length (≥10,000 bp)	555,531,269	409,213,261
Total length (≥25,000 bp)	553,048,228	407,729,606
Total length (≥50,000 bp)	549,835,103	403,929,324
# contigs	834	384
Largest contig	11,917,930	42,661,789
Total length	555,823,926	409,254,878
GC (%)	33.37%	33.88%
N50	2,284,547	21,796,443
N75	928,295	8,369,068
L50	61	7
L75	155	14
# N's per 100 kbp	0.10	0.78

**Table 2.** BUSCO (v.6) statistics for the initial raw draft genome and final, decontaminated assembly for *C. chalcites* based on the insecta_obd12 BUSCO library.

*C. chalcites* BUSCO statistics						
BUSCO	Complete	Single	Duplicated	Fragmented	Missing	*n*
Draft	98.6%	90.0%	8.5%	0.4%	1.0%	3114
Final	98.1%	95.2%	2.9%	0.5%	1.4%	3114

### *Phanaeus vindex* genome assembly

Initially, the raw *P. vindex* genome had a cumulative length of ∼553.8 Mbp and consisted of 3,595 contigs with an N50 of 11.91 Mbp. After decontamination and further removal of haplotype contigs, the final *P. vindex* nuclear genome had a cumulative length of ∼409.25 Mbp, comprising 384 contigs with a low L50 of 7, showing high contiguity. The N50 was 21.796 Mbp, and the GC content was 33.88%. All other QUAST statistics can be found in Table 1. BUSCO scores showed the final genome assembly to be 98.6% complete, with a single-copy completeness score of 95.8%, with 0.001% gaps (Table 3). For the final assembly, there was a 2.8% BUSCO duplication score, an improvement over the 6.6% duplication score of the initial draft assembly. The final genome sequencing coverage was ∼30×.

**Table 3.** BUSCO (v.6) statistics for the initial raw draft genome of *P. vindex* vs BUSCO statistics for the final, decontaminated assembly based on the insecta_obd12 BUSCO library.

*P. vindex* BUSCO statistics						
BUSCO	Complete	Single	Duplicated	Fragmented	Missing	*n*
Draft	98.7%	92.1%	6.6%	0.2%	1.1%	3114
Final	98.6%	95.8%	2.8%	0.2%	1.2%	3114

.

### RepeatMasker and repeat annotation

In *C. chalcites*, 69.96% (388,916,714 bp) of the genome was masked when we used our custom RepeatModeler library. For *P. vindex*, a much lower percentage of the genome was masked, with RepeatMasker masking 45.08% (184,507,231 bp) of the genome. The repetitive content of *C. chalcites* and *P. vindex* is comparable to the repetitive content found in other sequenced dung beetle genomes to date (Xu et al. 2025). Tables 4 and 5 show a detailed output of the classes of repetitive elements that were present in each of the genomes. For both genomes, unclassified repeats made up the majority of repetitive elements (28.51% for *C. chalcites* and 18.03% for *P. vindex*), followed by DNA transposons. Interestingly, *C. chalcites* had a larger percentage (26.84%) of its genome made up of DNA transposons than *P. vindex* (15.03%). Of the DNA transposon families, the hobo-activator family had a much larger presence in the *C. chalcites* genome, making up 7.31% of the DNA transposons vs only comprising 0.93% of the DNA transposons in *P. vindex* (Table 5).

**Table 4.** RepeatMasker statistics for *C. chalcites* and *P. vindex* using RepeatModeler libraries generated from each of the respective strains.

Repeat classes	*C. chalcites*	*P. vindex*
Bases masked	69.97%	45.08%
Retroelements	13.68%	9.73%
SINEs	0.00%	0.03%
LINESs	12.52%	7.36%
Penelope	1.57%	2.76%
LTR's	1.16%	2.35%
DNA transposons	26.84%	15.30%
Rolling circles	0.20%	0.87%
Unclassified	28.51%	18.03%
Small RNA	0.07%	0.21%
Satellites	0.03%	0.00%
Simple repeats	0.53%	0.79%
Low complexity	0.11%	0.18%

**Table 5.** Breakdown of the DNA transposon families in *C. chalcites* and *P. vindex*. The Hobo-Activator family has much higher representation in *C. chalcites* of 7.31% than in *P. vindex*, where it makes up <1% of the genome.

DNA transposons	*C. chalcites*	*P. vindex*
Hobo-Activator	7.31%	0.93%
Tc1-IS630-Pogo	11.35%	9.03%
EN-Spm	0.00%	0.00%
MuDR-IS905	0.00%	0.00%
PiggyBac	1.24%	1.44%
Tourist/Harbinger	0.29%	0.00%
Other	0.56%	0.02%

### Gene prediction

All transcriptomic libraries that were used for genome annotation passed QC after trimming for quality and removing adapters. For *C. chalcites*, 15 paired-read RNA-seq libraries were used for genome annotation, all passing FastQC with mean quality scores and mean per sequence phred scores > 38. For *P. vindex*, 17 paired-read libraries were used, all with mean quality phred scores > 35. For *C. chalcites*, BRAKER3 predicted 12,801 genes, with 15,817 coding DNA sequences (CDS). The BUSCO completeness score for *C. chalcites* gene annotations was 93.7%, with a single-copy score of 74.1% (Table 6). *P. vindex* had 12,252 genes and 15,120 CDS, with an average gene length of 8,779 bp. For *P. vindex* gene annotations, the BUSCO completeness score was 94.9%, with a single-copy score of 76.4% (Table 6). Other BRAKER3 gff statistics, generated by AGAT, can be found in Table 6. The gff and protein fasta files for both genomes can be found in Supplementary Files 1 (*C. chalcites*) and 2 (*P. vindex*). InterProScan annotated 12,865 genes with functional domains, encompassing 4,811 unique InterPro domains in *C. chalcites*. In *P. vindex*, InterProScan annotated 12,626 genes, encompassing 4,860 unique InterPro domains. The InteProScan TSV files for both genomes can also be found in Supplementary Files 1 and 2.

**Table 6.** Gene annotation statistics for BRAKER3 output for *C. chalcites* and *P. vindex*.

BRAKER3	*C. chalcites*	*P. vindex*
Number of genes	12,799	12,252
Number of transcripts	15,813	15,120
Number of CDS	15,813	15,120
Number of introns	69,081	70,036
Mean exons per transcript	5.4	5.6
Mean introns per transcript	4.4	4.6
Total gene length (bp)	173,425,152	109,888,172
Mean gene length (bp)	13,550	8,776
Total CDS length (bp)	23,958,570	23,957,038
Mean CDS length (bp)	1,515	1,584
Mean intron length (bp)	3,185	1,890
BUSCO with insecta_obd12 library on protein sequences, *n* = 3114		
Complete	93.7%	94.9%
Single-copy	74.1%	76.4%
Duplicated	19.5%	18.5%
Fragmented	1.0%	0.7%
Missing	5.3%	4.4%

Statistics were generated using AGAT.

### Orthologous gene comparisons

**Fig. 1.**
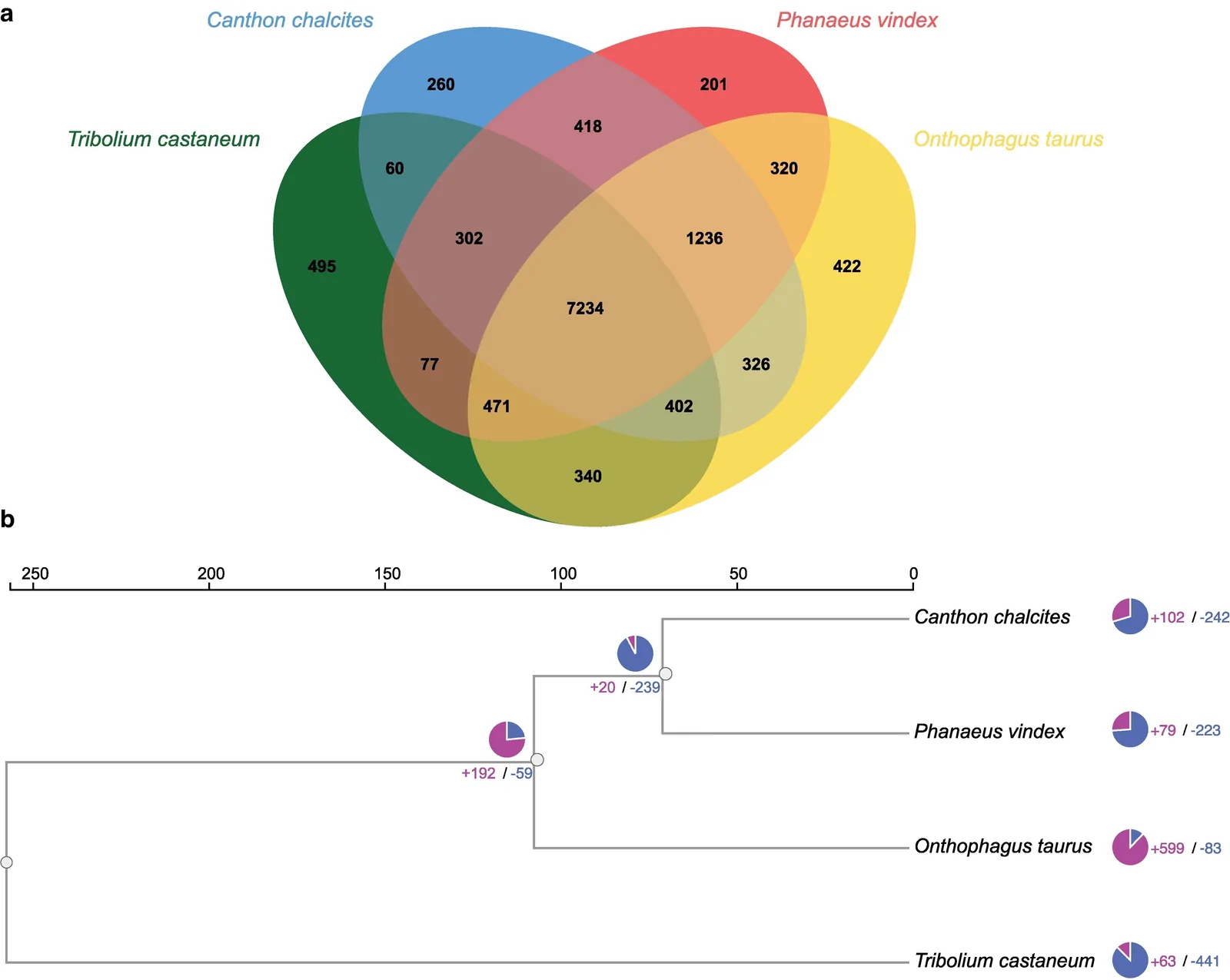
a) Ortholog CDS comparisons between *C. chalcites*, *P. vindex*, the dung beetle *O. taurus*, and a more distant Coleoptera member, *T. castaneum*. The Venn diagram shows the number of protein family clusters that overlap between the 4 species. Among the 4, there is a core set of 7,234 proteins. For the 3 dung beetle species, there are 1,236 core protein clusters that overlap. The orthologous analysis also shows that both *C. chalcites* and *P. vindex* have some unique proteins, with 418 clusters overlapping for the 2 species only. b) Evolutionary contractions/expansions analysis between the 4 Coleoptera species. The Venn diagram and evolutionary contraction tree were generated by the OrthoVenn3 software (Sun et al. 2023).

We ran 2 different types of comparisons with the predicted protein sequences from *C. chalcites* and *P. vindex*. For the orthologous protein analysis and the expansion and contraction analysis, we used the protein sequences of *C. chalcites*, *P. vindex*, the dung beetle *O. taurus* (refseq assembly GCF_036711975.1), and *T. castaneum* (refseq assembly GCA_000002335.3), a member of the Coleoptera order (Fig. 1a). Among the 4 species, we identified 7,234 conserved orthologous clusters. Within the 3 dung beetle species, 1,236 orthologous clusters were shared. The unique protein clusters for our 2 species of interest, *C. chalcites* and *P. vindex*, were 260 and 201, respectively.

**Fig. 2.**
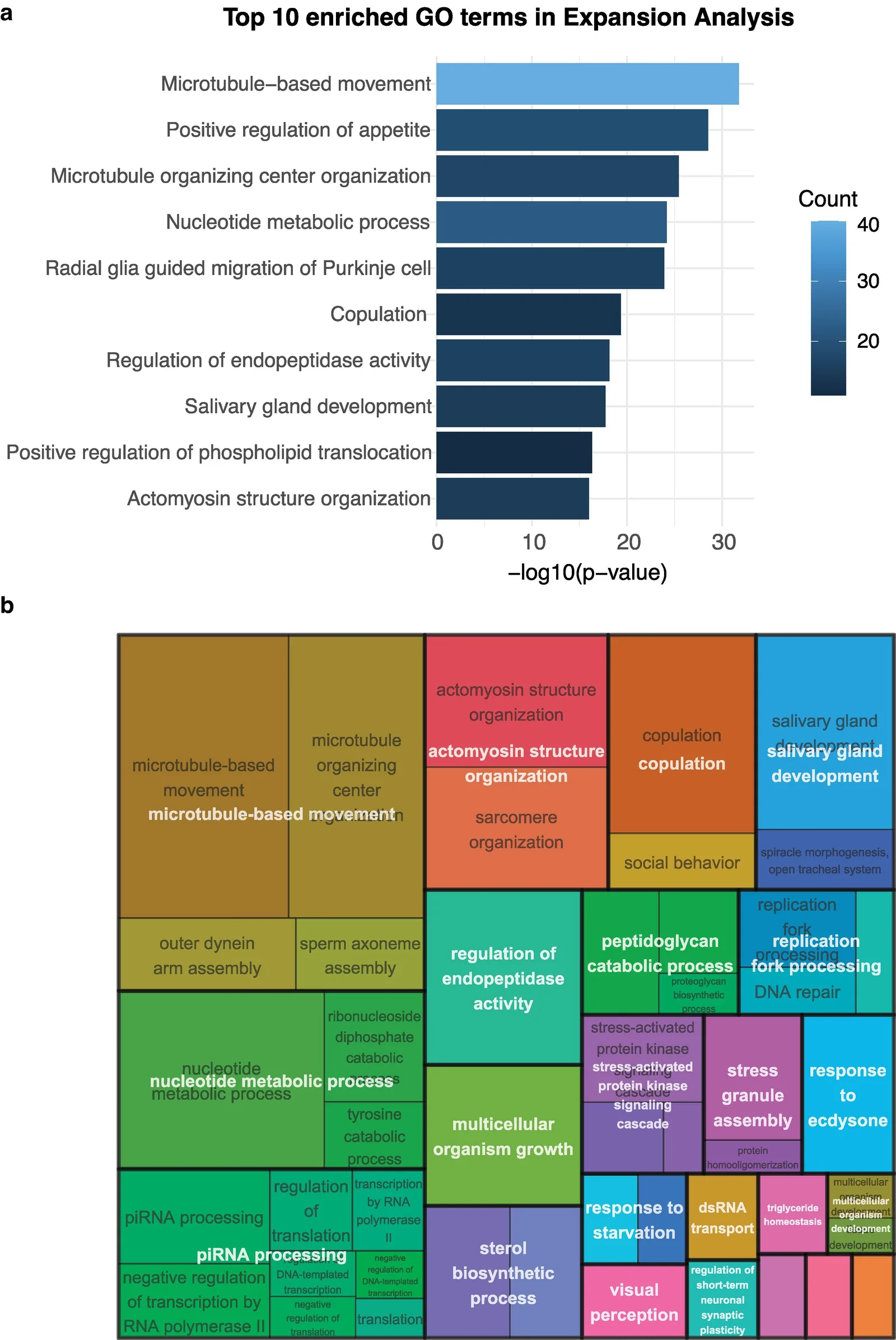
a) Top 10 GO-enriched terms in *C. chalcites* for the expansion analysis, shown in Fig. 1b. The order of significance is shown in descending log-transformed *P* values. b) Semantic similarity analysis done using rrvgo R software. The *Drosophila* database was used for mapping GO terms.

We also did an evolutionary analysis on the proteomes to see which protein families were expanded in *C. chalcites* and *P. vindex* relative to the 2 other Coleoptera species (Fig. 1b). *Canthon chalcites* had 105 gene cluster expansions and *P. vindex* had 79. For the expanded protein families in *C. chalcites* and *P. vindex*, GO enrichment analyses were calculated by OrthoVenn3 (Supplementary Tables 3 and 4). For *C. chalcites*, the top 3 GO-enriched terms were involved in transmembrane transport (GO:0055085, *P* < 0.001), radial glia-guided migration of Purkinje cell (GO:0021942, *P* < 0.001), and transcriptional regulation (GO:0006355, *P* < 0.001) (Fig. 2). When reduced for redundancy, rrvgo created 32 parent clusters out of the 52 GO terms associated with biological processes (Supplementary Table 5). Twenty-two GO terms did not map to the *Drosophila* GO database (Supplementary Table 6). The highest scoring parent cluster belonged to transmembrane transport (parent GO:0055085). In insects, transmembrane transporters are involved in a broad range of biological processes, including metabolic processes (Chaudhari et al. 2025), detoxification (Wu et al. 2019), the transportation of solutes (Denecke et al. 2020), hormones (Okamoto and Yamanaka 2021), and various other ions and molecules (Broehan et al. 2013), making transmembrane transporters important for responding to environmental stressors. However, many parent clusters in the rrvgo analysis for *C. chalcites* are also involved in development, reproduction, and signaling processes, such as wing disk development (GO:0035220), pigment cell differentiation (GO:0050931), larval somatic muscle development (GO:0007526), and various pathways important for growth that are grouped under the parent term calcium-mediated signaling (GO:0050848, GO:0050848, GO:0050848) (Supplementary Table 5). Other parent clusters are associated with eye development, such as rhabdomere development (child term grouped with wing disk development), visual behavior (GO:0007632), and visual perception (GO:0007691). In other dung beetle species, differences in wing shape and eye size are important for habitat partitioning (Tocco et al. 2019). In general, it seems that many of the expanded enriched GO terms in *C. chalcites* are involved in developmental processes and possibly environmental stress/acclimation (transmembrane transporters).

**Fig. 3.**
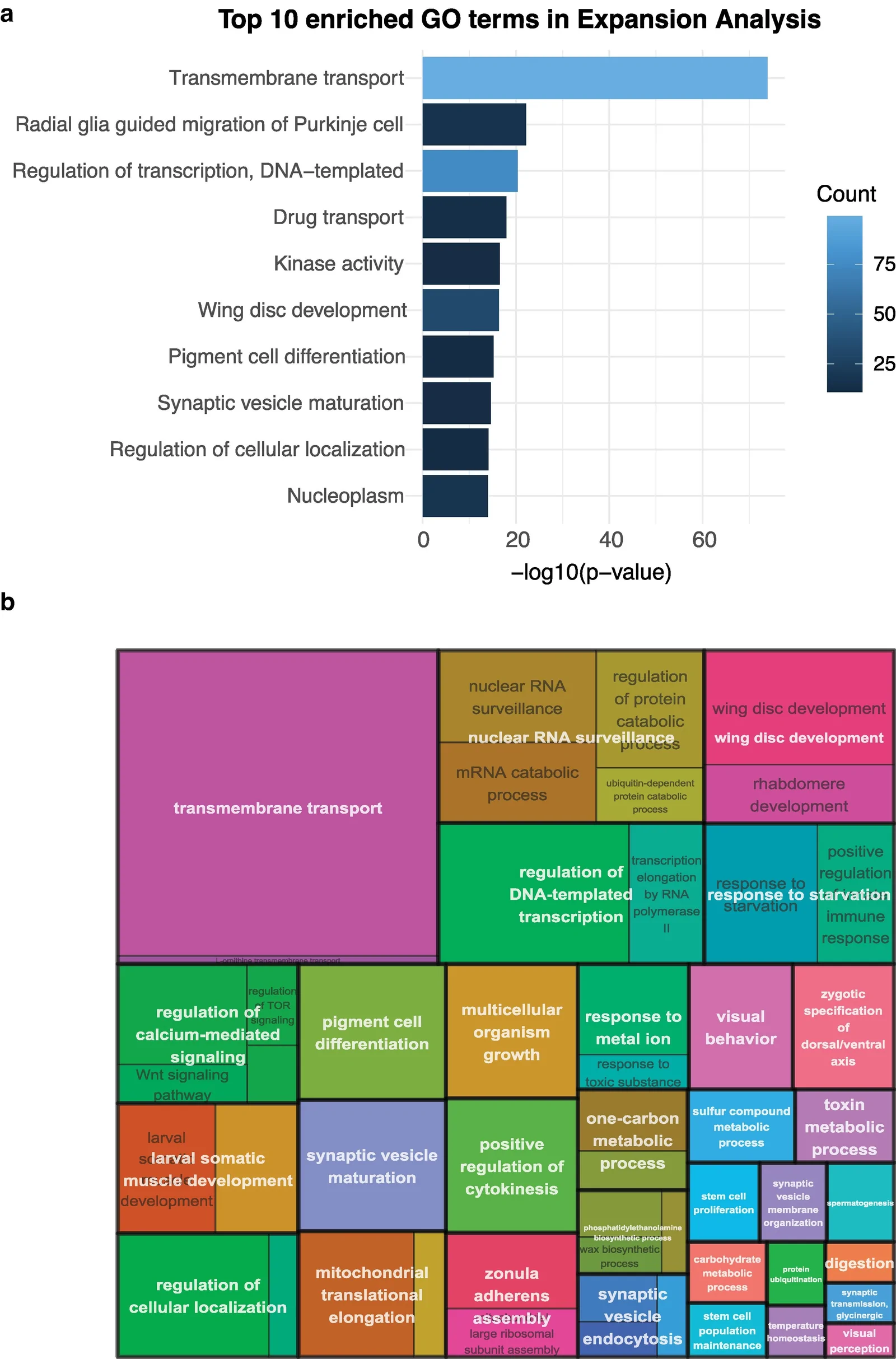
a) Top 10 GO-enriched terms in *P. vindex* for the expansion analysis, shown in Fig. 1b. The order of significance is shown in descending log-transformed *P* values. b) Semantic similarity analysis done using rrvgo R software. The *Drosophila* database was used for mapping GO terms.

For *P. vindex*, the top 3 most significantly enriched GO terms for the expansion analysis had to do with microtubule movement (GO:0007018, *P* < 0.001), microtubule organization (GO:0032100, *P* < 0.001), and appetite regulation (GO:0031023, *P* < 0.001) (Fig. 3). When we reduced the enriched GO terms for redundancy in *P. vindex*, rrvgo created 23 parent clusters out of the 54 GO terms (with biological process ontology) that mapped to the *Drosophila* GO database (Supplementary Table 7). Fifteen enriched GO terms for *P. vindex* did not map to the *Drosophila* database (Supplementary Table 8). For the GO terms that mapped, the highest scoring parent cluster belonged to microtubule-based movement and processes (Fig. 3; Supplementary Table 7). Microtubules serve various biological functions in insects and are involved in processes such as cytoskeletal development (Kim and Denlinger 2009), spermatogenesis (Mrinalini and Puniamoorthy 2021), and oocyte development (Stebbings and Hunt 1987). It has been found that microtubule abundance is involved in the cold response in the mosquito *Culex pipiens*, where low temperatures and diapause reduce microtubule abundance in the midgut and thoracic muscles (Kim and Denlinger 2009). However, microtubules are also involved in reproductive processes, and some of the GO terms (GO:0036158, GO:0007288) in the microtubule parent grouping are involved in outer dynein arm assembly and sperm axoneme assembly. Many other parent terms in the GO expansion enrichment analysis are also associated with reproductive, developmental, and mitotic processes, such as copulation (parent GO:0007620), piRNA processing (Santos et al. 2023) (parent GO:0034587), multicellular organism growth and development (parent GO:0007275), response to ecdysone (parent GO:0035075), and replication fork processing (parent GO:0031297) (Fig. 3). Other research using transcriptomes from the dung beetle *Catharsius molossus* also found novel reproductive genes not found in *O. taurus* (which also displays major–minor dimorphism) and more distantly related beetles, findings which reflected the trend found by other researchers that accessory glands in male insects seem to evolve rapidly (Mrinalini and Puniamoorthy 2021). Since male *P. vindex* beetles display major–minor dimorphism, a trait that is not universal among dung beetles, the *P. vindex* genome may provide additional genomic insight into the reproductive strategies of dung beetles (Killeffer et al. 2025) and more genomic insights into major–minor dimorphism.

### Conclusion

In this manuscript, we introduced 2 new dung beetle genome assemblies, both with high completeness scores. These 2 species were collected from eastern Tennessee and, based on our preliminary gene ortholog comparisons, will provide new genomic insights into dung beetle diversity, reproductive strategies, and evolution. Additionally, as these 2 species occupy temperate regions, their genomes may offer additional insights into behavior and thermal tolerance, important mechanisms of survival in these regions.

## Supplementary Material

jkag194_Supplementary_Data

## Data Availability

The genome assemblies for *P. vindex* and *C. chalcites* can be found on NCBI GenBank, under the BioProject ID's PRJNA1304933 and PRJNA1304937, respectively. Raw ccs fastq reads were also uploaded to NCBI SRA under the respective BioProject IDs. Supplementary Files 1 to 4 can be found on Figshare at https://doi.org/10.6084/m9.figshare.32440788. Scripts used for genome assembly and annotation can be found at https://github.com/microgs/Dung-Beetle-Assembly.git. The publically available genomes of *O.taurus* and *T.castaneum* that we used for comparisons in this manuscript be found on NCBI Genbank at accession numbers GCA_036711975.1 and GCA_000002335.3, respectively. Supplemental material available at *G3* online.

## References

[jkag194-B1] Dainat J . AGAT: Another Gff Analysis Toolkit to handle annotations in any GTF/GFF format. (Version v1.4.1). Zenodo. 10.5281/zenodo.3552717

[jkag194-B2] Bale JS, Hayward SAL. 2010. Insect overwintering in a changing climate. Journal of Experimental Biology. 213:980–994. 10.1242/jeb.037911. 20190123

[jkag194-B3] Broehan G, Kroeger T, Lorenzen M, Merzendorfer H. 2013. Functional analysis of the ATP-binding cassette (ABC) transporter gene family of *Tribolium castaneum*. BMC Genomics. 14:. 10.1186/1471-2164-14-6. PMC3560195 23324493

[jkag194-B4] Carter AW, Sheldon KS. 2020. Life stages differ in plasticity to temperature fluctuations and uniquely contribute to adult phenotype in dung beetles. Journal of Experimental Biology. 223:jeb227884. 10.1242/jeb.227884. 32917819

[jkag194-B5] Challis R, Richards E, Rajan J, Cochrane G, Blaxter M. 2020. BlobToolKit—interactive quality assessment of genome assemblies. G3 (Bethesda). 10:1361–1374. 10.1534/g3.119.400908. 32071071 PMC7144090

[jkag194-B6] Chaudhari BY, Pradhan AG, Joshi RS. 2025. Metabolic gatekeepers: dynamic roles of sugar transporters in insect metabolism and physiology. Insect Mol Biol. 34:1–18. 10.1111/imb.12963. 39394882

[jkag194-B7] Chen SF . 2023. Ultrafast one-pass FASTQ data preprocessing, quality control, and deduplication using fastp. IMeta. 2:e107. 10.1002/imt2.107. 38868435 PMC10989850

[jkag194-B8] Chen SF, Zhou YQ, Chen YR, Gu J. 2018. Fastp: an ultra-fast all-in-one FASTQ preprocessor. Bioinformatics. 34:884–890. 10.1093/bioinformatics/bty560. 30423086 PMC6129281

[jkag194-B103] deCastro-Arrazola I *et al*. 2022. A trait-based framework for dung beetle functional ecology. J Anim Ecol. 10.1111/1365-2656.13829. PMC10099951 36443916

[jkag194-B9] De Coster W, Rademakers R. 2023. NanoPack2: population-scale evaluation of long-read sequencing data. Bioinformatics. 39:btad311. 10.1093/bioinformatics/btad311. 37171891 PMC10196664

[jkag194-B10] Denecke SM et al 2020. The identification and evolutionary trends of the solute carrier superfamily in arthropods. Genome Biol Evol. 12:1429–1439. 10.1093/gbe/evaa153. 32681801 PMC7487162

[jkag194-B100] Fleming JM, Carter AW, Sheldon KS. 2021. Dung beetles show metabolic plasticity as pupae and smaller adult body size in response to increased temperature mean and variance. J Insect Physiol. 131. 10.1016/j.jinsphys.2021.104215. 33662376

[jkag194-B11] Flynn JM et al 2020. RepeatModeler2 for automated genomic discovery of transposable element families. Proc Natl Acad Sci U S A. 117:9451–9457. 10.1073/pnas.1921046117. 32300014 PMC7196820

[jkag194-B12] Gabriel L et al 2024. BRAKER3: fully automated genome annotation using RNA-seq and protein evidence with GeneMark-ETP, AUGUSTUS, and TSEBRA. Genome Res. 34:769–777. 10.1101/gr.278090.123. 38866550 PMC11216308

[jkag194-B13] Guan DF et al 2020. Identifying and removing haplotypic duplication in primary genome assemblies. Bioinformatics. 36:2896–2898. 10.1093/bioinformatics/btaa025. 31971576 PMC7203741

[jkag194-B14] Gurevich A, Saveliev V, Vyahhi N, Tesler G. 2013. QUAST: quality assessment tool for genome assemblies. Bioinformatics. 29:1072–1075. 10.1093/bioinformatics/btt086. 23422339 PMC3624806

[jkag194-B101] Holley JM, Andrew N.R. 2019. Experimental warming disrupts reproduction and dung burial by a ball-rolling dung beetle. Ecol Entomol. 44:206–221.

[jkag194-B15] Killeffer AT et al 2025. Testing for differences in metabolism among females and dimorphic males of four dung beetle species (Coleoptera: Scarabaeinae). Integrative Organismal Biology. 7:obaf031. 10.1093/iob/obaf031. 40896563 PMC12392089

[jkag194-B16] Kim D, Paggi JM, Park C, Bennett C, Salzberg SL. 2019. Graph-based genome alignment and genotyping with HISAT2 and HISAT-genotype. Nat Biotechnol. 37:907–915. 10.1038/s41587-019-0201-4. 31375807 PMC7605509

[jkag194-B17] Kim M, Denlinger DL. 2009. Decrease in expression of beta-tubulin and microtubule abundance in flight muscles during diapause in adults of *Culex pipiens*. Insect Mol Biol. 18:295–302. 10.1111/j.1365-2583.2009.00870.x. 19523062

[jkag194-B18] Kirkpatrick WH, Sheldon KS. 2022. Experimental increases in temperature mean and variance alter reproductive behaviours in the dung beetle. Biol Lett. 18:20220109. 10.1098/rsbl.2022.0109. 35857889 PMC9256084

[jkag194-B19] Li H . 2018. Minimap2: pairwise alignment for nucleotide sequences. Bioinformatics. 34:3094–3100. 10.1093/bioinformatics/bty191. 29750242 PMC6137996

[jkag194-B102] Macagno A *et al*. 2018. Adaptive maternal behavioral plasticity and developmental programming mitigate the transgenerational effects of temperature in dung beetles. Oikos. 127:1319–1329.

[jkag194-B21] Mamantov MA, Sheldon KS. 2023. Seasonality, distribution, and diversity of dung beetles (Coleoptera: Scarabaeidae: Scarabaeinae, Aphodiinae and Geotrupidae: Geotrupinae) in Great Smoky Mountains National Park, USA. Coleopt Bull. 77:285–295. 10.1649/0010-065X-77.3.285.

[jkag194-B22] Manni M, Berkeley MR, Seppey M, Zdobnov EM. 2021. BUSCO: assessing genomic data quality and beyond. Curr Protoc. 1:e323. 10.1002/cpz1.323. 34936221

[jkag194-B23] Marshall KE, Sinclair BJ. 2012. The impacts of repeated cold exposure on insects. Journal of Experimental Biology. 215:1607–1613. 10.1242/jeb.059956. 22539727

[jkag194-B24] Matthews BJ, Vosshall LB. 2020. How to turn an organism into a model organism in 10 ‘easy’ steps. Journal of Experimental Biology. 223:jeb218198. 10.1242/jeb.218198. 32034051 PMC7790198

[jkag194-B25] Moczek AP, Emlen DJ. 2000. Male horn dimorphism in the scarab beetle, *Onthophagus taurus*: do alternative reproductive tactics favour alternative phenotypes? Anim Behav. 59:459–466. 10.1006/anbe.1999.1342. 10675268

[jkag194-B26] Mrinalini CYK, Puniamoorthy N. 2021. Rapid genomic evolution drives the diversification of male reproductive genes in dung beetles. Genome Biol Evol. 13:evab172. 10.1093/gbe/evab172. 34426833 PMC8382682

[jkag194-B104] Nichols E *et al*. 2008. Ecological functions and ecosystem services provided by Scarabaeinae dung beetles. Biol Conserv. 141:1461–1474.

[jkag194-B27] Okamoto N, Yamanaka N. 2021. Transporter-mediated ecdysteroid trafficking across cell membranes: a novel target for insect growth regulators. J Pestic Sci. 46:23–28. 10.1584/jpestics.D20-071. 33746543 PMC7953032

[jkag194-B105] Oliveira RF, Taborsky M, Brockmann HJ. 2008. Alternative reproductive tactics: an integrative approach. Cambridge University Press. p. 1–22.

[jkag194-B28] Pertea G, Pertea M. 2020. GFF utilities: GffRead and GffCompare. F1000Res. 9:304. 10.12688/f1000research.23297.1. PMC7222033 32489650

[jkag194-B29] Pertea M et al 2015. StringTie enables improved reconstruction of a transcriptome from RNA-seq reads. Nat Biotechnol. 33:290–295. 10.1038/nbt.3122. 25690850 PMC4643835

[jkag194-B30] Quevillon E et al 2005. InterProScan: protein domains identifier. Nucleic Acids Res. 33:W116–W120. 10.1093/nar/gki442. 15980438 PMC1160203

[jkag194-B31] Santos D et al 2023. What are the functional roles of Piwi proteins and piRNAs in insects? Insects. 14:187. 10.3390/insects14020187. 36835756 PMC9962485

[jkag194-B32] Sayols S . 2023. Rrvgo: a Bioconductor package for interpreting lists of gene ontology terms. MicroPubl Biol. 2023:10.17912/micropub.biology.000811. 10.17912/micropub.biology.000811. PMC10155054 37151216

[jkag194-B33] Seppey M, Manni M, Zdobnov EM. 2019. BUSCO: assessing genome assembly and annotation completeness. In: Kollmar M, editor. Gene Prediction. Methods in Molecular Biology. vol 1962. Humana. p. 227–245. 10.1007/978-1-4939-9173-0_14. 31020564

[jkag194-B34] Sheldon KS, Padash M, Carter AW, Marshall KE. 2020. Different amplitudes of temperature fluctuation induce distinct transcriptomic and metabolomic responses in the dung beetle. Journal of Experimental Biology. 223:jeb233239. 10.1242/jeb.233239. 33139393

[jkag194-B35] Shen W, Le S, Li Y, Hu FQ. 2016. SeqKit: a cross-platform and ultrafast toolkit for FASTA/Q file manipulation. PLoS One. 11:e0163962. 10.1371/journal.pone.0163962. 27706213 PMC5051824

[jkag194-B36] Simao FA, Waterhouse RM, Ioannidis P, Kriventseva EV, Zdobnov EM. 2015. BUSCO: assessing genome assembly and annotation completeness with single-copy orthologs. Bioinformatics. 31:3210–3212. 10.1093/bioinformatics/btv351. 26059717

[jkag194-B37] Stebbings H, Hunt C. 1987. The translocation of mitochondria along insect ovarian microtubules from isolated nutritive tubes—a simple reactivated model. J Cell Sci. 88:641–648. 10.1242/jcs.88.5.641. 3503060

[jkag194-B38] Sun JH et al 2023. OrthoVenn3: an integrated platform for exploring and visualizing orthologous data across genomes. Nucleic Acids Res. 51:W397–W403. 10.1093/nar/gkad313. 37114999 PMC10320085

[jkag194-B39] Tarasov S, Dimitrov D. 2016. Multigene phylogenetic analysis redefines dung beetles relationships and classification (Coleoptera: Scarabaeidae: Scarabaeinae). BMC Evol Biol. 16:257. 10.1186/s12862-016-0822-x. 27899070 PMC5129633

[jkag194-B40] Tarasov S, Génier F. 2015. Innovative Bayesian and parsimony phylogeny of dung beetles (Coleoptera, Scarabaeidae, Scarabaeinae) enhanced by ontology-based partitioning of morphological characters. PLoS One. 10:e0116671. 10.1371/journal.pone.0116671. 25781019 PMC4363793

[jkag194-B41] Tocco C, Dacke M, Byrne M. 2019. Eye and wing structure closely reflects the visual ecology of dung beetles. J Comp Physiol A Neuroethol Sens Neural Behav Physiol. 205:211–221. 10.1007/s00359-019-01324-6. 30830308

[jkag194-B42] Uliano-Silva M et al 2023. MitoHiFi: a python pipeline for mitochondrial genome assembly from PacBio high fidelity reads. BMC Bioinformatics. 24:288. 10.1186/s12859-023-05385-y. 37464285 PMC10354987

[jkag194-B43] Wassmer T, Armstrong E. 2023. Population structure of *Phanaeus vindex* (Coleoptera: Scarabaeidae) in SE Michigan. J Insect Sci. 23:2. 10.1093/jisesa/iead050. PMC10317053 37399115

[jkag194-B44] Wu C, Chakrabarty S, Jin MH, Liu KY, Xiao YT. 2019. Insect ATP-binding cassette (ABC) transporters: roles in xenobiotic detoxification and Bt insecticidal activity. Int J Mol Sci. 20:2829. 10.3390/ijms20112829. 31185645 PMC6600440

[jkag194-B45] Xu X et al 2025. Chromosome-level genome assembly of the dung beetle Catharsius molossus (Coleoptera: Scarabaeidae). Sci Data. 12:1663. 10.1038/s41597-025-05765-4. 41120344 PMC12540882

